# Reference intervals for plasma IFN-α, TNF-α, IL-12p70, and IFN-γ by flow cytometry in healthy adults from eastern China: a single-center study

**DOI:** 10.3389/fimmu.2026.1806685

**Published:** 2026-05-14

**Authors:** Qian Liu, Linru Zhang, Xuanxuan Jiao, Yefu Sun, Fang Yang, Fumeng Yang

**Affiliations:** 1 Department of Laboratory Medicine, Affiliated Lianyungang Clinical College of Nantong University, Lianyungang, China; 2 Department of Laboratory Medicine, The Second People’s Hospital of Lianyungang Affiliated with Kangda College of Nanjing Medical University, Lianyungang, China; 3 Department of Laboratory Medicine, Affiliated Lianyungang Clinical College of Bengbu Medical University, Lianyungang, China; 4 Department of Laboratory Medicine, Lianyungang Clinical College of Xuzhou Medical University, Lianyungang, China; 5 Department of Laboratory Medicine, Chengcheng County Hospital, Weinan, China; 6 School of Medicine, Yangzhou Polytechnic University, Yangzhou, China

**Keywords:** cytokines, flow cytometry, IFN-α, IFN-γ, IL-12p70, reference intervals, TNF-α

## Abstract

**Background:**

Cytokines are essential regulators of immune homeostasis and play critical roles in both physiological immune modulation and pathological inflammatory processes. In this study, flow cytometry was applied to quantify plasma levels of interferon-α (IFN-α), tumor necrosis factor-α (TNF-α), interleukin-12p70 (IL-12p70), and interferon-γ (IFN-γ), aiming to establish reference intervals that may support clinical disease assessment and therapeutic decision-making.

**Methods:**

Between July and November 2025, 728 healthy adults from eastern China’s Jiangsu region were enrolled as reference subjects based on predefined eligibility criteria. Plasma levels of IFN-α, TNF-α, IL-12p70, and IFN-γ were measured via flow cytometry. The Kolmogorov–Smirnov test was used to evaluate data normality. Following the CLSI C28-A3 and WS/T 402-2024 guidelines, reference intervals for the four plasma cytokines in the overall population were derived using a nonparametric method covering the 2.5th–97.5th percentile range.

**Results:**

Plasma concentrations of four cytokines demonstrated non-Gaussian distributions. No significant differences were observed between sexes or among age groups (all P > 0.05), allowing combined analysis across all participants. The established reference intervals were as follows: IFN-α: 0.13–5.81 pg/mL, TNF-α: 0.60–4.89 pg/mL, IL-12p70: 0.24–5.44 pg/mL, and IFN-γ: 0.50–6.88 pg/mL.

**Conclusions:**

This study established flow cytometry-based reference intervals for plasma IFN-α, TNF-α, IL-12p70, and IFN-γ in healthy adults from Jiangsu Province, eastern China. These intervals provide a reliable reference for assessing immune status, guiding the management of immune-related diseases, and supporting laboratories in the adoption or establishment of reference intervals.

## 1 Introduction

Cytokines are low–molecular weight, soluble polypeptides that act as key intercellular signaling mediators, secreted by immune cells as well as various tissue-resident cells (1). Among them, interferon-α (IFN-α), tumor necrosis factor-α (TNF-α), interleukin-12p70 (IL-12p70), and interferon-γ (IFN-γ) play central roles in immune regulation and are involved in multiple pathological processes (2–5). These cytokines participate in fundamental physiological immune responses and contribute to the development of a broad spectrum of diseases, including malignancies, infectious disorders, inflammatory conditions, and coronary artery disease (6–10). Accordingly, accurate *in vitro* measurement of these cytokines is essential for assessing immune status and supporting clinical diagnosis and treatment strategies. In clinical practice, reference intervals constitute a critical framework for interpreting laboratory results and guiding medical decision-making (11). However, well-established and validated reference intervals for plasma cytokines are still limited, and many clinical laboratories rely on manufacturer-provided ranges without independent verification (12). Since reference intervals may differ according to geographic location, population characteristics, and analytical methodologies, careful evaluation is required before their clinical application (13). The application of unsuitable reference intervals may result in misinterpretation of test results, thereby increasing the risk of diagnostic inaccuracies and inappropriate therapeutic interventions (13). Therefore, it is essential for clinical laboratories to develop and validate method-specific and population-based reference intervals for clinically important cytokines, including IFN-α, TNF-α, IL-12p70, and IFN-γ.

In this study, plasma levels of IFN-α, TNF-α, IL-12p70, and IFN-γ were measured in a healthy adult population using flow cytometric techniques. The distributions of these cytokines were analyzed with respect to age and sex. In accordance with the recommendations of the Clinical and Laboratory Standards Institute (CLSI) C28-A3 and WS/T 402-2024 guidelines (14, 15), reference intervals specific to a healthy cohort from Jiangsu Province in eastern China were established and reported. These rigorously derived reference intervals are expected to enhance the clinical utility of cytokine testing in preventive health assessments, diagnostic decision-making, and longitudinal therapeutic monitoring.

## 2 Materials and methods

### 2.1 Study population

**Figure 1:**
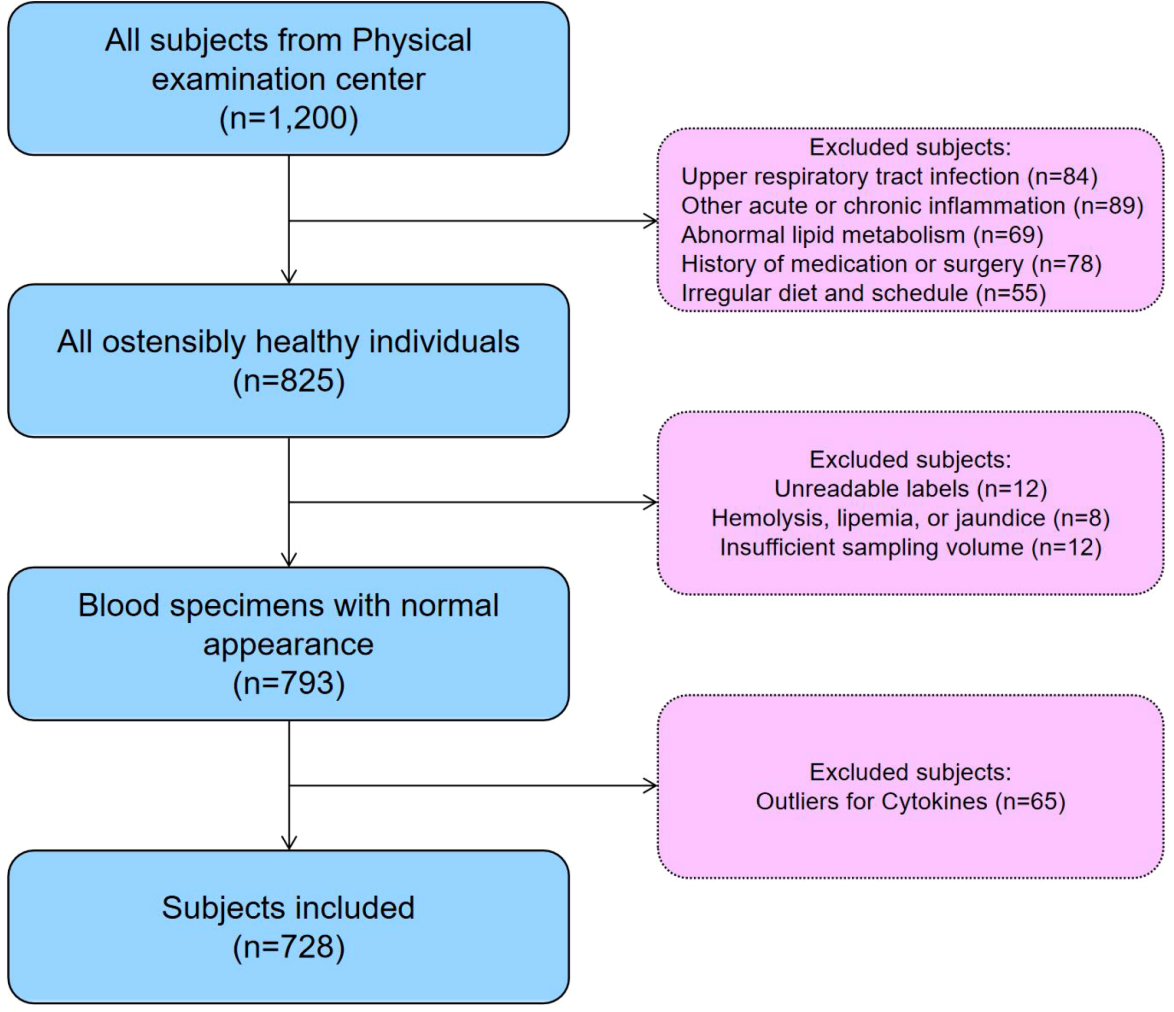
Flowchart of reference individual recruitment.

From July to November 2025, a total of 1,200 individuals undergoing routine health examinations at the Physical Examination Center of the Second People’s Hospital of Lianyungang were prospectively recruited using simple random sampling. After applying predefined inclusion and exclusion criteria, 728 participants met the eligibility requirements and were included in the final analysis. The study cohort consisted of 364 men and 364 women, with ages ranging from 20 to 79 years. A detailed flow diagram illustrating the participant recruitment and selection process is shown in **Figure 1**.

#### 2.1.1 Eligibility criteria

Eligible participants were required to meet the following inclusion criteria: (1) age between 20 and 79 years with a body mass index (BMI) of 18.5–27.9 kg/m²; (2) normotensive status, defined as systolic blood pressure of 90–140 mmHg and diastolic blood pressure of 60–90 mmHg; (3) routine hematological and biochemical parameters within established reference limits, including white blood cell count (WBC), C-reactive protein (CRP), indices of hepatic and renal function, fasting glucose, and lipid profiles; (4) negative serologic screening for hepatitis B virus, hepatitis C virus, syphilis, and human immunodeficiency virus; and (5) no clinically relevant abnormalities detected on physical examination, chest radiography, electrocardiography, or abdominal ultrasonography.

#### 2.1.2 Exclusion criteria

Participants were excluded if any of the following conditions were present: (1) recent surgery or current use of medications; (2) recent history of blood donation, transfusion, or significant blood loss; (3) long-term or occupational exposure to physical or chemical hazards, including ionizing radiation, benzene, or lead; (4) Chronic alcohol overuse (i.e., heavy drinking occurring on ≥4 days per week on average), or excessive alcohol consumption during the 14-day period before enrollment (with daily pure alcohol intake >60 g for males and >40 g for females), or severe tobacco smoking (>20 cigarettes/day); (5) pregnancy or breastfeeding; (6) hematological disorders, such as anemia, leukemia, or thrombocythemia; (7) active allergic diseases, including urticaria, asthma, or allergic dermatitis; (8) respiratory diseases, including acute infections, tuberculosis, or chronic obstructive pulmonary disease (COPD); (9) disorders of the urinary system; (10) gastrointestinal diseases; (11) autoimmune or rheumatic conditions; (12) thyroid-related disorders; (13) confirmed parasitic infections; or (14) a previous diagnosis of malignancy.

This study was reviewed and approved by the Medical Ethics Committee of the Second People’s Hospital of Lianyungang (approval No. 2022K043). Written informed consent was obtained from all participants prior to enrollment.

### 2.2 Study design

In accordance with the CLSI C28-A3 and WS/T 402-2024 guidelines (14, 15), participants were initially stratified by sex. When a statistically significant difference between sexes was identified (P < 0.05), age-based partitioning was subsequently conducted within each sex using the following categories: 20–29, 30–39, 40–49, 50–59, and ≥60 years. In contrast, if no significant sex-related difference was observed (P ≥ 0.05), data from all participants were combined and stratified solely by age using the same predefined intervals. Based on the final stratification scheme, plasma reference intervals for IFN-α, TNF-α, IL-12p70, and IFN-γ were then calculated.

### 2.3 Specimen collection

Participants were advised to maintain their usual dietary habits and physical activity levels for three days prior to blood collection. After an overnight fast, venous blood (14 mL) was obtained in the morning. The collected samples were distributed into two 2-mL EDTA-K_2_ anticoagulated tubes and two 5-mL serum tubes. These specimens were used for cytokine analysis as well as for routine hematological, biochemical, and immunological examinations.

### 2.4 Instruments and reagents

Plasma concentrations of IFN-α, TNF-α, IL-12p70, and IFN-γ were determined using a DxFLEX flow cytometer (Beckman Coulter, Brea, CA, USA) in combination with commercially available assay kits (Qingdao Raisecare Biotechnology Co., Ltd., Qingdao, China; lot number 240511), strictly following the manufacturer’s instructions. White blood cell counts and C-reactive protein (CRP) levels were assessed using a BC-7500CRP automated hematology analyzer (Shenzhen Mindray Biomedical Electronics Co., Ltd., Shenzhen, China).

Serum biochemical parameters, including alanine aminotransferase (ALT; lot AUZ3365), aspartate aminotransferase (AST; lot AUZ3727), urea (lot AUZ4364), creatinine (Crea; lot 2574), glucose (GLU; lot AUZ3936), total cholesterol (TC; lot AUZ4150), and triglycerides (TG; lot AUZ4052), were measured on an AU5800 automated chemistry analyzer (Beckman Coulter, Brea, CA, USA). All analytical instruments underwent routine calibration and maintenance prior to testing. Internal quality control procedures and external quality assessment programs were implemented throughout the study to ensure that analytical performance consistently met established quality requirements.

### 2.5 Analytical performance characteristics of cytokine assays

In this study, a commercially available assay kit was used. The manufacturer had comprehensively validated the analytical performance of the methodology in accordance with guidelines such as YY/T 1789.3-2022, CNAS-GL037, CNAS-GL047, and WS/T 408-2024 (16–19). Subsequently, we performed verification on the in-house testing platform based on the manufacturer’s specifications. The verified performance parameters included limit of blank (LoB), limit of detection (LoD), lower limit of quantification (LLoQ), intra- and inter-assay precision, trueness, linearity, and reportable range. The verification results for all parameters were consistent with the manufacturer’s specifications (detailed findings are provided in **Supplementary Table 1**). In addition, a rigorous daily quality control procedure was implemented to continuously monitor the analytical performance of the testing system and to ensure its stability throughout the study period.

### 2.6 Cytokine assay principle and procedure

Cytokine measurements were performed using a direct sandwich immunoassay format. Briefly, cytokines present in plasma samples or calibration standards were first captured by fluorescent microspheres coated with cytokine-specific antibodies. A biotinylated secondary antibody was then added to bind the captured analyte, forming a sandwich immune complex. After incubation with streptavidin conjugated to phycoerythrin, fluorescence signals corresponding to each bead population were detected by flow cytometry. Cytokine concentrations were calculated based on fluorescence intensity. All assay steps were carried out in strict accordance with the manufacturer’s instructions.

### 2.7 Reference intervals validation

In accordance with the recommendations of WS/T 402-2024 (15), this study validated the established reference intervals using a large independent validation cohort (n = 60). For each cytokine, an additional 60 reference individuals who met the identical inclusion and exclusion criteria were enrolled. The validation cohort was prospectively collected independently of the reference population, and all procedures for specimen collection, transportation, and processing were strictly consistent with those applied to the reference population. Outliers were excluded and replaced to ensure a final sample size of 60. Validation was considered successful if at least 90% of the results (i.e., ≥54 out of 60) fell within the established reference intervals. If this criterion was not met, a second round of validation was conducted using another group of 60 individuals. The reference intervals were considered validated if the ≥90% requirement was achieved in the second assessment; otherwise, validation was considered unsuccessful.

### 2.8 Procedures for outlier identification and processing

Outliers were identified and removed using Tukey’s method (15). Q1 (25th percentile), Q3 (75th percentile), and interquartile range (IQR = Q3 − Q1) were calculated to determine the lower and upper boundaries: lower boundary = Q1 − 1.5 × IQR, upper boundary = Q3 + 1.5 × IQR. Data points outside these boundaries were considered outliers and excluded. This process was repeated iteratively until all data fell within the boundaries. If the sample size dropped below 120 after outlier removal, additional participants were enrolled to reach 120. The main goal of outlier removal is to reduce the influence of extreme values on percentiles, thereby enhancing the scientific soundness and standardization of the statistical outcomes.

### 2.9 Statistical analyzes

Statistical analyzes were conducted using SPSS software (version 19.0; IBM Corp.). Data distribution was assessed for normality with the Kolmogorov–Smirnov test. Normally distributed variables are presented as mean ± standard deviation, whereas non-normally distributed data are expressed as median (M) with IQR. Group comparisons were performed using nonparametric tests, including the Mann–Whitney U test for two-group comparisons and the Kruskal–Wallis test followed by Dunn’s *post hoc* test for multiple groups. Whether reference intervals needed separate groupings for sex and age was judged using effect size estimates. Reference intervals were defined using the nonparametric method covering the 2.5th–97.5th percentile range. A two-sided P value <0.05 was considered statistically significant.

## 3 Results

### 3.1 Participant characteristics and baseline laboratory data

Based on the predefined eligibility criteria, a total of 728 individuals were included in the reference population. Demographic characteristics including sex, age, smoking status, alcohol consumption and body mass index (BMI) were recorded for all participants. In addition, routine laboratory parameters such as WBC, CRP, ALT, AST, Urea, Crea, GLU, TC, and TG were measured and analyzed. A comprehensive summary of the demographic and laboratory characteristics of the study cohort is presented in **Supplementary Table 2**.

### 3.2 Correlation analysis between cytokines and routine indicators

**Figure 2:**
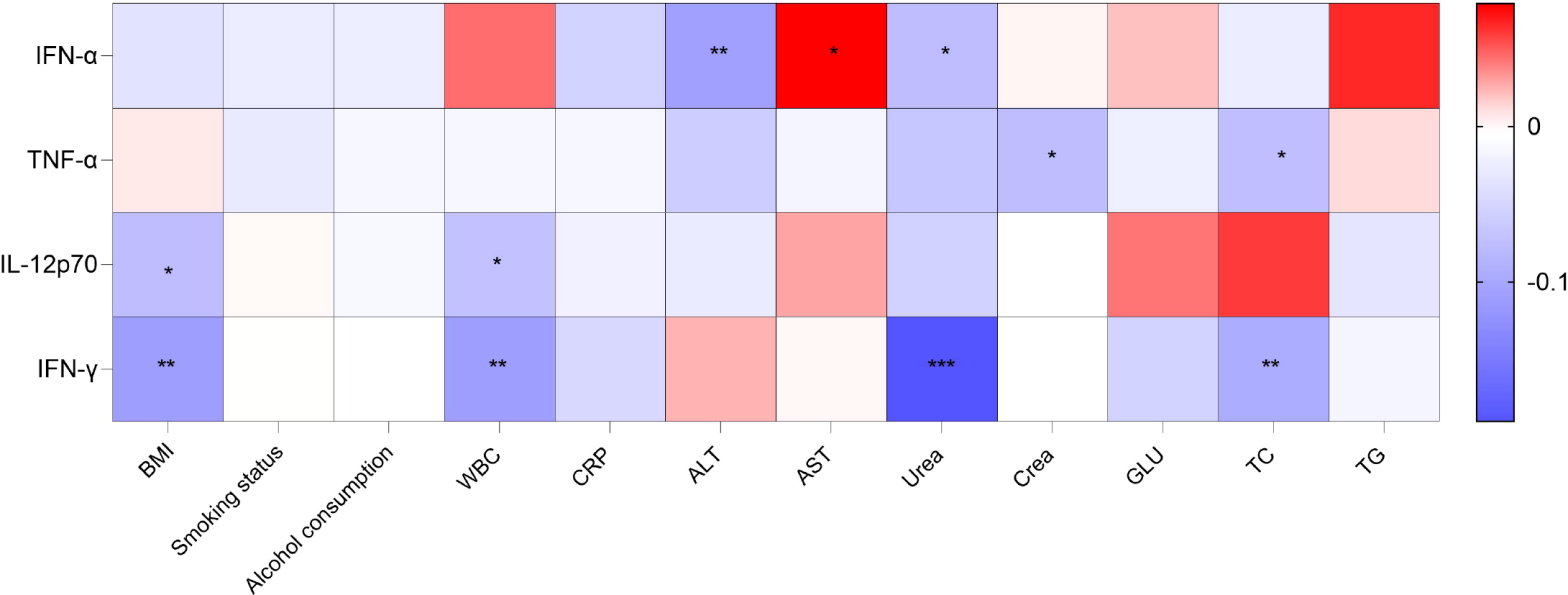
Correlation heatmap between cytokines and routine indicators. Spearman correlation coefficients are shown. Blue indicates negative correlation, red indicates positive correlation, white indicates zero correlation. *p < 0.05, **p < 0.01, ***p < 0.001.

Spearman’s rank correlation analysis was conducted to evaluate the associations between four cytokines (IFN-α, TNF-α, IL-12p70, and IFN-γ) and routine laboratory parameters (BMI, smoking status, alcohol consumption, WBC, CRP, ALT, AST, Urea, Crea, GLU, TC, and TG). The results demonstrated weak correlations between the cytokines and each of the laboratory indicators, with all correlation coefficients falling below 0.3. The detailed findings are shown in **Figure 2**.

### 3.3 Sex- and age-related distribution of plasma IFN-α, TNF-α, IL-12p70, and IFN-γ

**Figure 3:**
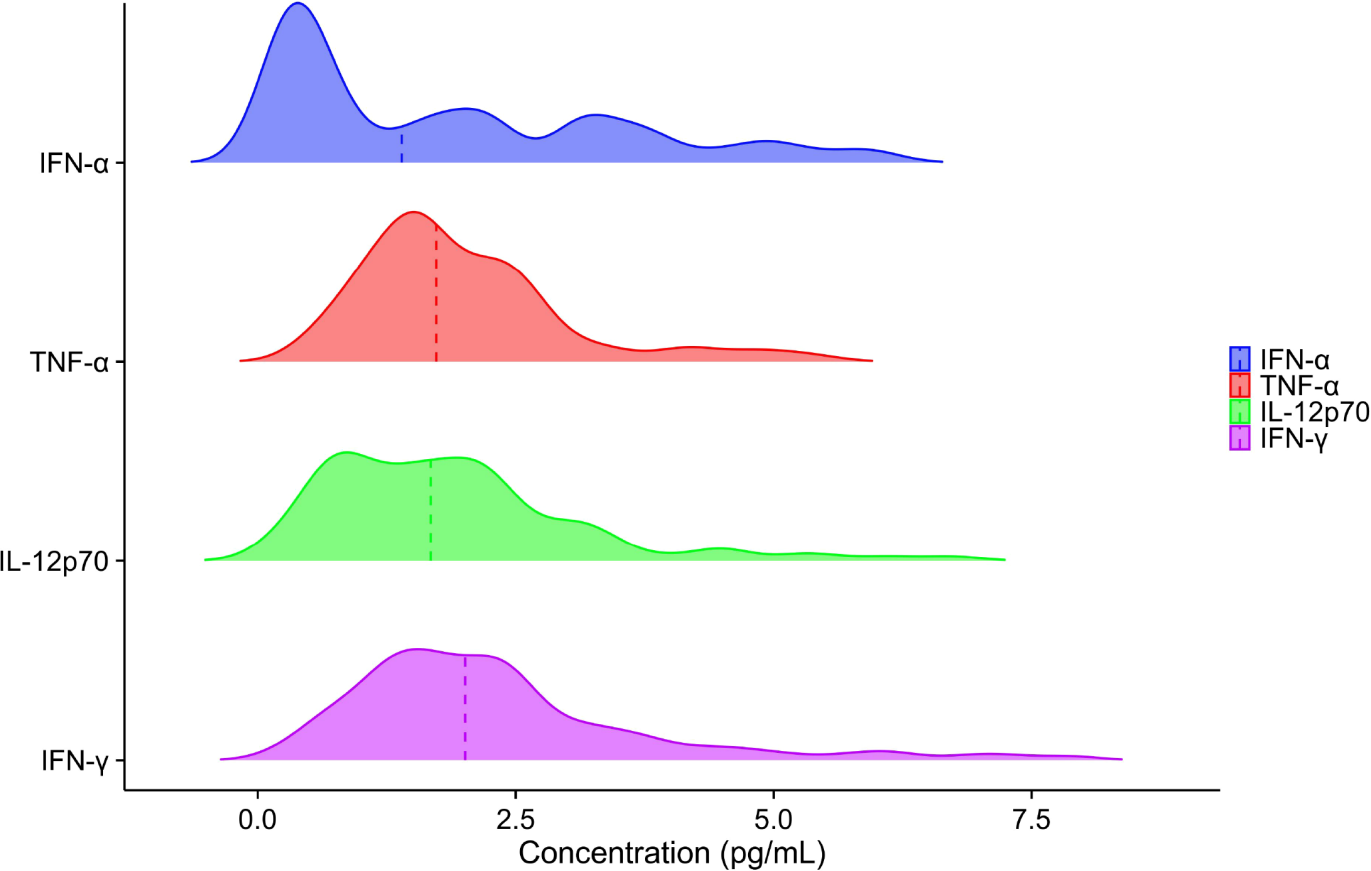
Distribution of plasma IFN-α, TNF-α, IL-12p70 and IFN-γ levels. The P-values from the Kolmogorov-Smirnov test were all less than 0.05.

**Figure 4:**
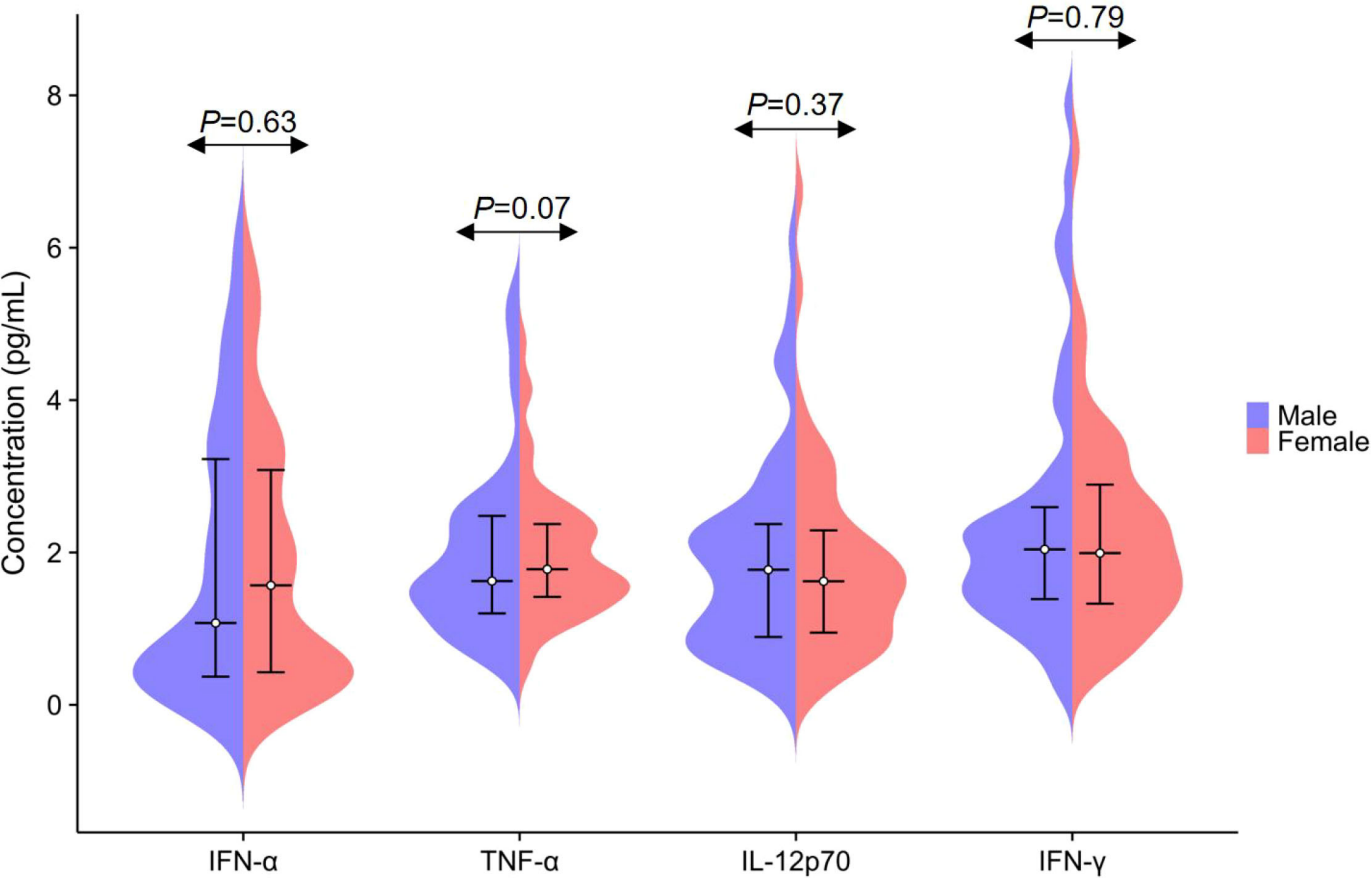
Comparison of plasma IFN-α, TNF-α, IL-12p70 and IFN-γ levels based on gender. The levels of IFN-α, TNF-α, IL-12p70, and IFN-γ are presented as median and interquartile range.

Normality was assessed using the Kolmogorov–Smirnov test, which revealed that the plasma concentrations of IFN-α, TNF-α, IL-12p70, and IFN-γ in the reference population did not follow a Gaussian distribution (**Figure 3**). Comparative analysis between male and female participants showed no statistically significant differences in the levels of any of the four cytokines (all P > 0.05; **Table 1**; **Figure 4**). Subsequently, participants were stratified into five age categories, and intergroup comparisons likewise indicated no significant differences in plasma IFN-α, TNF-α, IL-12p70, or IFN-γ concentrations across age groups (all P > 0.05; **Table 2**). In addition, effect size statistics were employed to guide decisions on stratification by sex or age. Specifically, Cohen’s d was used for sex-based comparisons, and the correlation coefficient (r) was applied for age-related analyzes. The results showed that all Cohen’s d values were below 0.2 and all r values were below 0.1, indicating consistently small effect sizes for both factors. Accordingly, stratification by sex or age was not considered necessary. Detailed effect size estimates are presented in **Table 3**.

**Table 1:** Comparison of plasma IFN-α, TNF-α, IL-12p70 and IFN-γ between gender groups.

Analytes	Total (n=728)	Gender groups		P-value
		Male (n=364)	Female (n=364)	
IFN-α (pg/mL)	1.40 (0.40-3.14)	1.08 (0.37-3.23)	1.57 (0.42-3.08)	0.63
TNF-α (pg/mL)	1.73 (1.33-2.44)	1.63 (1.20-2.48)	1.78 (1.41-2.38)	0.07
IL-12p70 (pg/mL)	1.68 (0.91-2.36)	1.78 (0.89-2.38)	1.62 (0.94-2.30)	0.37
IFN-γ (pg/mL)	2.01 (1.35-2.73)	2.04 (1.39-2.60)	1.99 (1.32-2.91)	0.79

IFN-α, Interferon-α; TNF-α, Tumor necrosis factor-α; IL-12p70, Interleukin-12p70; IFN-γ, Interferon-γ. The indicators of IFN-α, TNF-α, IL-12p70 and IFN-γ were represented as the median (M) and interquartile range (IQR).

**Table 2:** Comparison of plasma IFN-α, TNF-α, IL-12p70 and IFN-γ between age groups.

Analytes	Age groups					P-value
	20-29 years (n=126)	30-39 years (n=147)	40-49 years (n=140)	50-59 years (n=133)	≥60 years (n=182)	
IFN-α (pg/mL)	0.82 (0.35-3.10)	1.77 (0.40-3.00)	0.71 (0.34-3.04)	1.57 (0.43-3.85)	1.42 (0.47-3.05)	0.12
TNF-α (pg/mL)	1.75 (1.18-2.53)	1.61 (1.43-2.28)	1.92 (1.31-2.31)	1.94 (1.47-2.61)	1.71 (1.20-2.47)	0.33
IL-12p70 (pg/mL)	1.77 (0.89-2.38)	1.54 (0.89-2.20)	1.68 (0.66-2.21)	1.78 (0.71-2.92)	1.55 (1.08-2.60)	0.05
IFN-γ (pg/mL)	2.10 (1.51-3.86)	1.97 (1.26-2.62)	1.93 (1.29-2.54)	2.15 (1.66-2.99)	2.11 (1.24-3.04)	0.08

IFN-α, Interferon-α; TNF-α, Tumor necrosis factor-α; IL-12p70, Interleukin-12p70; IFN-γ, Interferon-γ. The indicators of IFN-α, TNF-α, IL-12p70 and IFN-γ were represented as the median (M) and interquartile range (IQR).

**Table 3:** Effect sizes of sex or age on plasma cytokine concentrations.

Parameter	Cytokines	Cohen’s d */r value**	Evaluation results
Gender	IFN-α	<0.01	Small effect
	TNF-α	0.02	Small effect
	IL-12p70	0.07	Small effect
	IFN-γ	0.10	Small effect
Age	IFN-α	0.04	Small effect
	TNF-α	0.01	Small effect
	IL-12p70	0.07	Small effect
	IFN-γ	0.03	Small effect

IFN-α, Interferon-α; TNF-α, Tumor necrosis factor-α; IL-12p70, Interleukin-12p70; IFN-γ, Interferon-γ.

*d < 0.2 indicates a small effect; 0.20 ≤ d < 0.50 indicates a small to medium effect; 0.50 ≤ d < 0.80 indicates a medium effect; d ≥ 0.80 indicates a large effect.

**r < 0.10 indicates a small effect; 0.10 ≤ r < 0.30 indicates a small to medium effect; 0.30 ≤ r < 0.50 indicates a medium effect; r ≥ 0.50 indicates a large effect.

**Figure 5:**
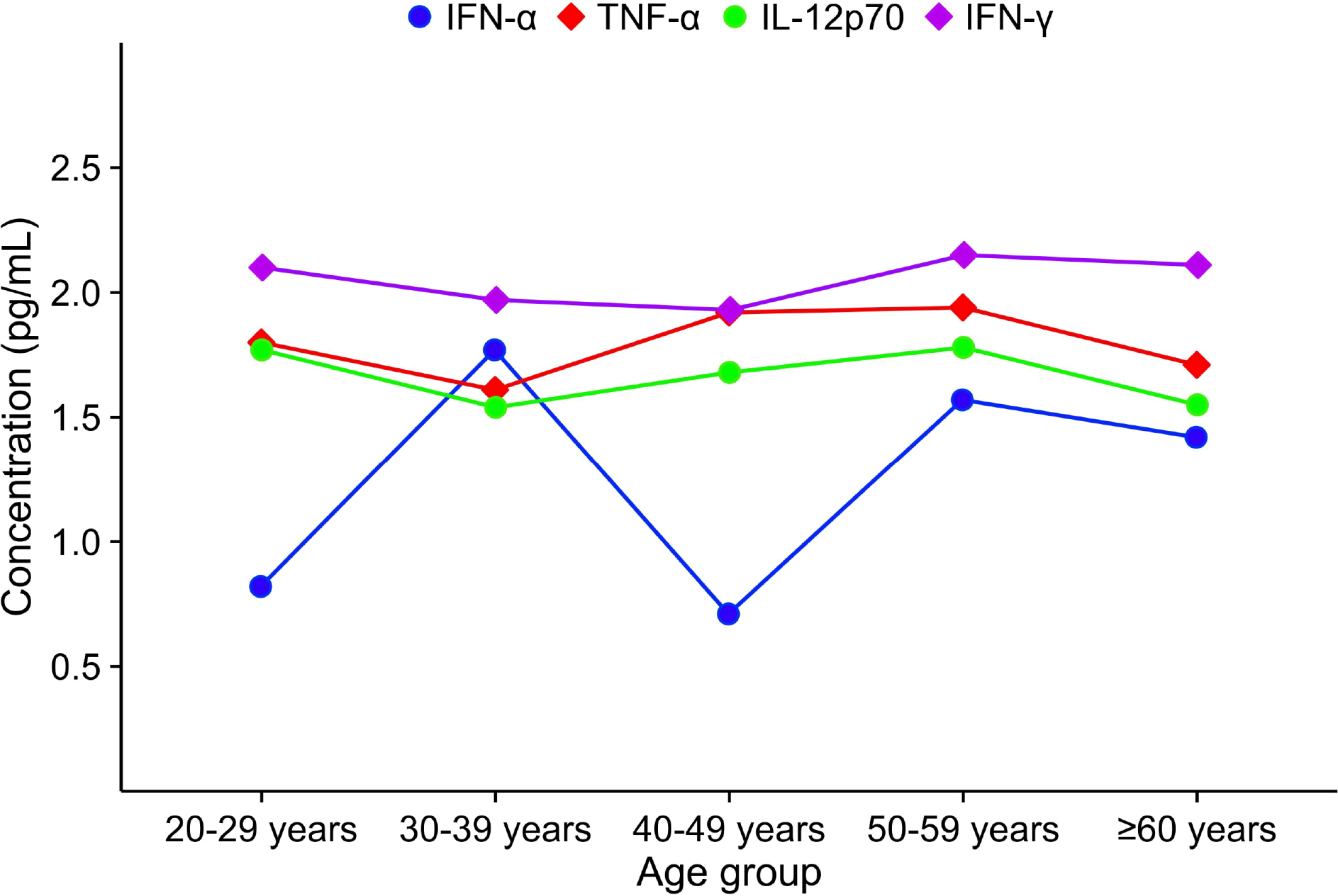
The trend of plasma IFN-α, TNF-α, IL-12p70, and IFN-γ across different age groups. Plasma levels of IFN-α, TNF-α, IL-12p70, and IFN-γ in different age groups are all expressed as median.

Additionally, we applied line graphs to visually depict the trends of the four indicators across different age groups. IFN-α exhibited a W-shaped pattern, whereas TNF-α, IL-12p70, and IFN-γ showed a pattern characterized by an initial decrease, followed by an increase, and then another decrease. The specific trends are presented in **Figure 5**.

### 3.4 Establishment of reference intervals for plasma IFN-α, TNF-α, IL-12p70, and IFN-γ

In the absence of significant differences among sex- or age-stratified subgroups, the whole study population was pooled as a unified reference sample for interval estimation. Following the recommendations of CLSI C28-A3 and WS/T 402-2024, reference intervals were calculated nonparametrically across the 2.5th–97.5th percentile range. Flow cytometric measurement yielded the following reference intervals for the eastern Chinese population: IFN-α, 0.13–5.81 pg/mL; TNF-α, 0.60–4.89 pg/mL; IL-12p70, 0.24–5.44 pg/mL; and IFN-γ, 0.50–6.88 pg/mL. Corresponding data are summarized in **Table 4**.

**Table 4:** Distribution of plasma IFN-α, TNF-α, IL-12p70 and IFN-γ based on all subjects.

Analytes (pg/mL)	Distribution percentile (n=728)				
	P_2.5_ (90% CI)*	P_25_ (90% CI)	P_50_ (90% CI)	P_75_ (90% CI)	P_97.5_ (90% CI)
IFN-α	0.13 (0.12-0.15)	0.40 (0.36-0.43)	1.40 (0.99-1.62)	3.14 (3.05-3.24)	5.82 (5.49-5.91)
TNF-α	0.60 (0.51-0.66)	1.33 (1.26-1.40)	1.73 (1.65-1.80)	2.44 (2.35-2.50)	4.89 (4.74-5.07)
IL-12p70	0.24 (0.16-0.33)	0.91 (0.86-0.98)	1.68 (1.60-1.77)	2.36 (2.28-2.44)	5.44 (5.18-6.05)
IFN-γ	0.50 (0.36-0.56)	1.35 (1.29-1.42)	2.01 (1.93-2.12)	2.73 (2.60-2.97)	6.88 (6.20-7.21)

IFN-α, Interferon-α; TNF-α, Tumor necrosis factor-α; IL-12p70, Interleukin-12p70; IFN-γ, Interferon-γ; P_2.5_, 2.5th percentile; P_25_, 25th percentile; P_50_, 50th percentile; P_75_:75th percentile; P_95_, 95th percentile; P_97.5_, 97.5th percentile; CI, confidence interval.

*The 2.5th percentile of the plasma cytokine concentration was below LLoD; it is recommended that results be reported as “< LLoD”.

### 3.5 Validation of the reference intervals

An independent validation set comprising 60 apparently healthy individuals, matched for sex and spanning the age range of 20–79 years, was randomly selected from the source population. Validation analysis showed that more than 90% of cytokine measurements in this cohort were within the proposed reference intervals for IFN-α, TNF-α, IL-12p70, and IFN-γ (**Table 5**). These findings confirm the reliability and clinical applicability of the established reference intervals.

**Table 5:** Validation of established reference intervals.

Indicators	Reference intervals (pg/mL)	Number of reference individuals (n)	Proportion of results in the reference interval (%)	Evaluation criteria (%)	Conclusion (Pass or fail)
IFN-α	0.13-5.81	60	100.0	≥90.0	Pass
TNF-α	0.60-4.89	60	95.0	≥90.0	Pass
IL-12p70	0.24-5.44	60	95.0	≥90.0	Pass
IFN-γ	0.50-6.88	60	100.0	≥90.0	Pass

IFN-α, Interferon-α; TNF-α, Tumor necrosis factor-α; IL-12p70, Interleukin-12p70; IFN-γ, Interferon-γ.

## 4 Discussion

Cytokine measurement is commonly performed using analytical platforms such as enzyme-linked immunosorbent assay (ELISA), chemiluminescent immunoassays, and flow cytometry (20–22). Compared with other methods, flow cytometry provides notable advantages for multiplex cytokine analysis, including minimal sample volume requirements, high analytical throughput, robust precision, and the ability to simultaneously quantify multiple targets within a single assay (23, 24). These features have led to its widespread adoption in routine clinical laboratory practice. In the present study, plasma levels of IFN-α, TNF-α, IL-12p70, and IFN-γ were measured using a flow cytometry–based platform in strict accordance with the manufacturer’s instructions. To our knowledge, this represents the first assessment of these cytokines in a healthy adult population from Jiangsu Province in eastern China. By analyzing data from this single-center cohort, we aimed to enhance the clinical utility of these immunological markers and establish region-specific reference intervals.

Following rigorous inclusion and exclusion criteria, 728 individuals were ultimately identified as qualified reference subjects in this study. Our analysis revealed weak correlations between the four cytokines (IFN-α, TNF-α, IL-12p70, and IFN-γ) and routine laboratory parameters. Nevertheless, it is worth noting that factors such as heavy smoking, heavy drinking, and prolonged alcohol consumption were excluded from this study; consequently, the confounding influence on the cytokine profiles might be underestimated. Moreover, the distribution levels of these cytokines in the apparently healthy population were all non-normally distributed. A key objective was to evaluate the potential influence of demographic variables. After stratifying the participants by sex and dividing them into five age groups, appropriate nonparametric statistical analyzes were performed. The results revealed no significant differences in the levels of the four cytokines between sex groups or across age categories. Moreover, the effect size estimates for the influence of sex or age on cytokine concentrations consistently indicated small effects. These findings suggest that, within the examined adult population, the concentrations of these immune mediators are largely independent of sex and age, thereby supporting the establishment of common reference intervals. Additionally, we further applied line graphs to visually illustrate the trend changes of the four indicators across different ages. Previous investigations have reported partially concordant results. Lan et al. (25) analyzed serum interferon-γ, tumor necrosis factor-α, interleukin-2, interleukin-4, interleukin-6, interleukin-10, and interleukin-17A in 100 reference individuals using flow cytometry and found no statistically significant differences between males and females. A study by Li et al. (26), which included 126 healthy American individuals aged 18 to 64 years, also revealed no age- or sex-related effects on IFN-γ and TNF-α. Together with our findings, this suggests that the distribution patterns of these cytokines may be generalizable across different populations. In contrast, Huang et al. (27), who measured interleukin-1β, interleukin-6, interleukin-8, interleukin-10, and tumor necrosis factor-α in 156 ostensibly healthy subjects by chemiluminescence, observed a significant sex-related difference only for tumor necrosis factor-α, with higher concentrations in males. More recently, Xie et al. (28) reported significant sex-based differences in twelve cytokines, including IFN-α, TNF-α, IL-12p70, and IFN-γ; however, the relatively limited sample size of 299 participants precluded further age-stratified analyzes. In another study, Miwa et al. (29) assessed serum cytokine levels in a population of 157 Japanese individuals, and observed a more extensive age/sex effect on cytokine distribution in this group. The potential reasons for this difference may include: (a) divergent entry criteria—the 60-80 age range in Miwa’s work was determined only via medical history interviews without laboratory validation; (b) sample size discrepancies—the reference population comprised 157 subjects, limiting subgroup analysis power; and (c) marked differences in genetic background among populations. In summary, our findings largely align with those of previous studies, although certain inconsistencies exist. A common drawback of earlier investigations is their limited sample size, which restricts a robust exploration of age-and sex-related variations in cytokine levels. By contrast, the current investigation systematically examined the distributions of IFN-α, TNF-α, IL-12p70, and IFN-γ across both sex and age strata in a larger, well-defined regional cohort. This design substantially strengthens the statistical robustness and population representativeness of the derived reference intervals, thereby enhancing their applicability in stratified clinical assessment and decision-making.

Clinical laboratories have traditionally relied on established reference intervals because of their convenience and ease of implementation in routine practice (30, 31). However, the scientific validity of applying uniform reference intervals is increasingly being questioned. A key limitation of such generalized intervals is their potential mismatch with the demographic characteristics of local patient populations. Biological and sociodemographic variables, including ethnicity, sex, and age, are well recognized as important determinants of physiological biomarker distributions (30, 31). Consequently, the uncritical adoption of externally derived reference intervals without local verification may increase the risk of diagnostic misclassification. In response to this challenge, the harmonization and standardization of reference interval establishment have become central concerns in laboratory medicine, prompting extensive methodological research and professional consensus-building efforts (32–34). This global emphasis on precision has also been reflected in national initiatives. Notably, the National Health Commission of China has developed evidence-based reference intervals for commonly measured analytes through large-scale multicenter population studies, providing a standardized framework intended to support regional customization (35, 36). In light of the above research background and strategy, and following the recommendations of CLSI C28-A3 and WS/T 402-2024 (14, 15), we established reference intervals for plasma IFN-α, TNF-α, IL-12p70, and IFN-γ in healthy adults from Jiangsu Province. A nonparametric approach was applied using two-sided percentiles (2.5th to 97.5th percentile range), with due consideration of the clinical relevance of these cytokines. It should be emphasized that, although low concentrations of cytokines are of limited clinical significance, appropriate attention must still be given from the perspective of analytical performance characteristics. In particular, when cytokine levels approach or fall below the LLoQ, laboratories are advised to report the result as “<LLoQ” and to integrate the finding with clinical symptoms or other biomarkers for comprehensive assessment. This practice helps avoid overinterpretation in clinical decision-making that might arise from methodological limitations. Subsequently, in accordance with the approach recommended in WS/T 402-2024 (15), we validated the established reference intervals using an independent large-sample cohort (n=60), and the validation results met the required criteria. Compared with previous reports, our findings show certain discrepancies. For example, Zhang et al. (37), in their evaluation of a flow cytometry platform, reported manufacturer-supplied reference intervals for IFN-α at or below 8.5 pg/mL, TNF-α at or below 16.5 pg/mL, IL-12p70 at or below 3.4 pg/mL, and IFN-γ at or below 23.1 pg/mL. The upper bounds for IFN-α, TNF-α, and IFN-γ were notably higher than those identified in the present analysis. Similarly, Lan et al. (25) proposed serum reference intervals for TNF-α ranging from 5.723 to 12.878 ng/mL and for IFN-γ ranging from 2.950 to 4.697 ng/mL, both substantially higher than the corresponding upper limits observed in our study, although an opposite trend for IFN-γ has also been reported elsewhere (27). The discrepancies between the aforementioned findings and those of the present study may be attributed to several underlying mechanisms, including: 1) Effects of coagulation: Thrombin generation in serum activates monocytes and platelets, promoting the release of cytokines like interleukin-6 and interleukin-8, whereas anticoagulants (EDTA) in plasma inhibit coagulation, reducing cellular activation and cytokine release; 2) Role of platelets: Platelets in serum release cytokines (especially increasing interleukin-8) during coagulation, while platelet activation in plasma is relatively lower; 3) Differences in cell-matrix contact time: Serum separation requires a clotting time (≥30 minutes), increasing cell-serum contact and potentially altering cytokine levels, whereas cells in plasma remain suspended before centrifugation, allowing for more controlled contact time; 4) Differences in matrix stability: Certain cytokines (e.g., interleukin-8) in serum may be elevated due to cellular activation during coagulation, while cytokines in plasma are more stable and less affected by pre-analytical variability (1, 38). Accordingly, we emphasize that when establishing or adopting cytokine reference intervals, laboratories must carefully consider the specimen matrix, as reference intervals derived from serum and plasma are not interchangeable. Even greater divergence was observed in the study by Adedeji et al. (39), who used high-performance liquid chromatography in a Nigerian population and reported an IL-12 reference interval from 0.49 to 1.02 ng/L, with an upper limit far below that observed in our cohort. In addition, Xie et al. (28), who established sex-specific reference intervals for twelve cytokines, reported values that differed to varying extents from those obtained in the current study. These discrepancies are most plausibly attributed to differences in study populations. The present cohort was drawn from a coastal region of eastern China that is characterized by distinct genetic backgrounds, dietary patterns with relatively high seafood consumption, culinary traditions, and environmental exposures. Such region-specific lifestyle and environmental factors are known to modulate baseline inflammatory status and immune system activity. Collectively, these comparisons highlight a central principle that reference intervals are intrinsically population- and location-dependent. Evidence from prior investigations (25, 28, 37, 39), together with the findings of this study, clearly indicates that reference intervals cannot be assumed to be universally applicable. Accordingly, clinical laboratories should prioritize the development and verification of reference intervals tailored to their own population characteristics, analytical platforms, and pre-analytical conditions. The adoption of this population-centered approach is essential to ensure that laboratory results provide accurate, contextually relevant, and clinically reliable benchmarks to support informed medical decision-making.

Several limitations of the present study warrant consideration. First, this investigation was conducted at a single center, and the reference individuals were predominantly recruited from coastal regions of eastern China. As a result, the geographical generalizability of the findings may be constrained, and laboratories in other regions should apply the proposed reference intervals with appropriate caution. Second, the study population was limited to adults aged 20 to 79 years, and pediatric as well as adolescent groups were not represented. Therefore, the established reference intervals cannot be extrapolated to infants, children, or adolescents. Third, although the sample size met the minimum guideline requirement of 120 individuals per subgroup, the overall cohort size was relatively modest. The high cost of flow cytometry limited the feasibility of enrolling a larger number of participants. Future studies should aim to validate and optimize these reference intervals using multicenter, large-scale datasets and to expand inclusion across all age groups in order to enhance their generalizability and clinical applicability.

## 5 Conclusions

In summary, this study established reference intervals for plasma IFN-α, TNF-α, IL-12p70, and IFN-γ in healthy adults from eastern China using flow cytometry. These reference intervals provide reliable data to support the evaluation of immune status and the clinical management of immune-related diseases. Furthermore, they offer valuable guidance for laboratories in the appropriate adoption or establishment of reference intervals.

## Data Availability

The original contributions presented in the study are included in the article/**Supplementary Material**. Further inquiries can be directed to the corresponding author.

## References

[1] Feng X Zou W Li P Guo K Ma Y Hu G . Comparability evaluation of serum and plasma cytokine levels by multiplex bead-based flow cytometry. Clin Chim Acta. (2025) 575:120351. doi: 10.1016/j.cca.2025.120351. PMID: 40354961

[2] Jancsura MK Helsabeck NP Anderson CM Conley YP Hubel CA Roberts JM . Identifying the timing and type of inflammatory markers for potential prediction of preeclampsia in women with obesity. Hypertens Preg. (2025) 44:2492084. doi: 10.1080/10641955.2025.2492084. PMID: 40247440 PMC12079614

[3] Ren Z Ma X Liu Y Zhang Y Chen Y Sun C . Alterations in plasma cytokine profiles in generalized myasthenia gravis following different immunotherapeutic regimens. Front Neurol. (2025) 16:1728767. doi: 10.3389/fneur.2025.1728767. PMID: 41426979 PMC12714632

[4] Wu X Song HH Xu GR Li RY Ye XB . Serum cytokine profiles in patients with myasthenia gravis. Front Neurol. (2025) 16:1611673. doi: 10.3389/fneur.2025.1611673. PMID: 40689326 PMC12272165

[5] Dong Y Tang Y Li Y Cao P Xu G Zhu R . Role of peripheral cytokines and orbitofrontal cortex subregion structure in schizophrenia agitation. Sci Rep. (2025) 15:14125. doi: 10.1038/s41598-025-99033-5. PMID: 40269239 PMC12019167

[6] Zhang Z Zhou BH Hu L Li MC Chang D Dou X . Relationship between biologic therapy and cytokine levels in patients with inflammatory arthritis. Med (Balt). (2025) 104:e42953. doi: 10.1097/MD.0000000000042953. PMID: 40550053 PMC12187313

[7] Zhang Y Shen WX Li P Chen MB Zhou LN . Serum interleukin levels predict occurrence of acute radiation pneumonitis and overall survival in thoracic tumours. Clin Invest Med. (2025) 48:29–38. doi: 10.3138/cim-2024-0262. PMID: 40131214

[8] Wang R Yin G Guo W Li N Zhang Y Chen X . Analysis of Th1/Th2 cytokine profile and clinical characteristics of patients with head and neck squamous cell carcinoma. Biomol BioMed. (2024) 24:1776–84. doi: 10.17305/bb.2024.10783. PMID: 38920620 PMC11496867

[9] Liu S Wang C Guo J Yang Y Huang M Li L . Serum cytokines predict the severity of coronary artery disease without acute myocardial infarction. Front Cardiovasc Med. (2022) 9:896810. doi: 10.3389/fcvm.2022.896810. PMID: 35651907 PMC9149173

[10] Bi Q Zhu J Zheng J Xu Q Chen J Zhang Lc . Blood inflammatory markers and cytokines in COVID-19 patients with bacterial coinfections. Immun Inflammation Dis. (2024) 12:e70105. doi: 10.1002/iid3.70105. PMID: 39692539 PMC11653711

[11] Zhang GM Wang T Gu B . Establishing reference interval of serum CA-125 for the healthy Han population in China. BMC Cancer. (2026) 26(1):200. doi: 10.1186/s12885-026-15558-6. PMID: 41527076 PMC12888479

[12] Sepiashvili L Alli Z Bohn MK Hall A Karin A Murata K . Complex biological patterns of soluble cytokines and CD163 in childhood necessitating age-specific reference intervals for evidence-based clinical interpretation. Clin Biochem. (2021) 98:35–41. doi: 10.1016/j.clinbiochem.2021.09.004. PMID: 34509468 PMC9433181

[13] Guven B Benice I Acikgoz B Can M . The reference intervals of PT, INR and APTT tests on the Cobas analyzer in Turkish pediatric population. Scand J Clin Lab Invest. (2026) 86(1):36–41. doi: 10.1080/00365513.2025.2611810. PMID: 41503963

[14] Clinical and Laboratory Standards Institute . Defining, establishing, and verifying reference intervals in the clinical laboratory; approved guideline. In: Wayne: PA, CLSI C28-A3, 3rd ed. Wayne: Clinical and Laboratory Standards Institute (2010). doi: 10.6028/nist.fips.11-3-1991

[15] National Health Commission of the People's Republic of China . WS/T 402-2024 clinical laboratory test project reference range. Beijing, China: Standard Press (2024). doi: 10.32388/8u3751

[16] National Medical Products Administration . *In vitro* diagnostic test systems—Performance evaluation method—Part 3: limit of detection and limit of quantitation (Standard no. YY/T 1789.3-2022). Beijing: China Standard Press (2022). doi: 10.32388/sqcoma

[17] China National Accreditation Service for Conformity Assessment (CNAS) . Guidance on the verification of quantitative measurement procedures used in the clinical chemistry (Standard no. CNAS-GL037). Beijing: CNAS (2019). doi: 10.32388/lyn5cc

[18] China National Accreditation Service for Conformity Assessment (CNAS) . Medical laboratories—Guidance on the verification of result comparability among quantitative examination procedures (Standard no. CNAS-GL047). Beijing: CNAS (2021). doi: 10.32388/d6t5zu

[19] National Health Commission of the People's Republic of China . Guideline for the verification of analytical performance of quantitative examination procedures (standard no. WS/T 408-2024). Beijing: China Standard Press (2024).

[20] Labossiere EH Gonzalez-Diaz S Enns S Lopez P Yang X Kidane B . Detectability of cytokine and chemokine using ELISA, following sample-inactivation using Triton X-100 or heat. Sci Rep. (2024) 14:26777. doi: 10.1038/s41598-024-74739-0. PMID: 39500912 PMC11538312

[21] Yang X Zhao H Liu X Xie Q Zhou X Deng Q . The relationship between serum cytokine levels and the degree of psychosis and cognitive impairment in patients with methamphetamine-associated psychosis in Chinese patients. Front Psychiatry. (2020) 11:594766. doi: 10.3389/fpsyt.2020.594766. PMID: 33362607 PMC7759545

[22] Sfredo ADS Silva MABD Melo LVL França DCH Dantas GL Santos WBD . Hormonal and cytokine imbalances promote a proinflammatory profile in institutionalized elderly. Brain Sci. (2025) 15:1310. doi: 10.3390/brainsci15121310. PMID: 41440106 PMC12730809

[23] Boss K Hagen J Constans M Goetz C Kalyuzhny AE . Comparing flow cytometry and ELISpot for detection of IL-10, IL-6, and TNF alpha on human PBMCs. Methods Mol Biol. (2024) 2768:87–103. doi: 10.1007/978-1-0716-3690-9_6. PMID: 38502389

[24] Tovar Acero C Ramírez-Montoya J Velasco MC Avilés-Vergara PA Ricardo-Caldera D Duran-Frigola M . IL-4, IL-10, CCL2 and TGF-β as potential biomarkers for severity in Plasmodium vivax malaria. PloS NeglTrop Dis. (2022) 16:e0010798. doi: 10.1371/journal.pntd.0010798. PMID: 36178979 PMC9555658

[25] Lan X Li ZM Zhang MZ . Reference value of serum inflammatory cytokines in normal population from Zhengzhou. J Zhengzhou Univ (Med Sci). (2018) 53:341–3. doi: 10.13705/j.issn.1671-6825.2017.10.053

[26] Li Y Yi JS Russo MA Rosa-Bray M Weinhold KJ Guptill JT . Normative dataset for plasma cytokines in healthy human adults. Data Brief. (2021) 35:106857. doi: 10.1016/j.dib.2021.106857. PMID: 33665253 PMC7900339

[27] Huang Y Zhang L Wang F Wei W . Preliminary study on adult reference intervals for interleukin-1β, interleukin-6, interleukin-8, interleukin-10 and tumor nec-rosis factor α in serum. Chin J Clin Lab Sci. (2018) 36:549–51. doi: 10.13602/j.cnki.jcls.2018.07.19

[28] Xie H Zhang G Xia Y Pan J Shen H Xia M . Establishment of reference intervals for serum TNF-α, IFN-α, IL-1β, IL-2, IL-4, IL-5, IL-6, IL-8, IL-10, IL-12p70, IL-17 and IFN-γ in healthy Chinese adults using flow cytometry. J Immunol Methods. (2026) 547:114034. doi: 10.1016/j.jim.2026.114034. PMID: 41512996

[29] Miwa T Shimizu Y Ito T Inomata N Inoue E Sueki H . Impact of age and gender on serum cytokine and chemokine levels: Analysis of 157 healthy subjects across consecutive age groups. Int Arch Allergy Immunol. (2026), 1–21. doi: 10.1159/000551338. PMID: 41802125

[30] Liu Q Xia Z Huang T Yang F Wang X Yang F . Establishment of reference intervals for plasma IL-2, IL-4, IL-5, and IL-17A in healthy adults from the Jiangsu region of eastern China using flow cytometry: A single-center study. Cytokine. (2024) 179:156594. doi: 10.1016/j.cyto.2024.156594. PMID: 38581867

[31] Yang D Su Z Zhao M . Big data and reference intervals. Clin Chim Acta. (2022) 527:23–32. doi: 10.1016/j.cca.2022.01.001. PMID: 34999059

[32] Waggoner D Thoren K Maynard R Twum K Korpi-Steiner N . Evaluation of serum kappa free light chain reference interval using a new stabilized calibrator. J Appl Lab Med. (2026) 11(3):599–605. doi: 10.1093/jalm/jfaf208. PMID: 41543034

[33] Liu Q Wang H Shen S Yang F Han Z Liu E . Establishing reference intervals for serum glutathione reductase in healthy adults. Arch Med Sci. (2025) 21:1397–404. doi: 10.5114/aoms/199536. PMID: 41078950 PMC12509819

[34] Lauffer P Heinen CA Goorsenberg AWM Malekzadeh A Henneman P Heijboer AC . Analysis of serum free thyroxine concentrations in healthy term neonates underlines need for local and laboratory-specific reference interval: A systematic review and meta-analysis of individual participant data. Thyroid. (2024) 34:559–65. doi: 10.1089/thy.2023.0562. PMID: 38563802

[35] The National Health Commission of the People’s Republic of China . WS/T 404.10-2021 Reference intervals for common clinical biochemistry tests-Part 10: Serum triiodothyronine, thyroxine, free triiodothyronine, free thyroxine and thyroid stimulating hormone. Beijing: China Standard Press (2023). doi: 10.32388/2ths52

[36] The National Health Commission of the People’s Republic of China . Reference intervals for common clinical biochemistry tests-Part 9: Serum C-reactive protein, prealbumin, transferrin, β2-microglobulin. Beijing: China Standard Press (2019).

[37] Zhang YL Gao YD Tan MJ . A performance evaluation of multiplex microsphere flow immunofluorescence assays for the measurement of 12 cytokines. Labell Immunoassays Clin Med. (2022) 29:2131–6. doi: 10.11748/bjmy.issn.1006-1703.2022.12.030

[38] Yang X Arceo T Fischer SK . Effect of delayed blood centrifugation on cytokine quantitation in serum and plasma. Bioanalysis. (2025) 17:913–21. doi: 10.1080/17576180.2025.2544521. PMID: 40791022 PMC12369616

[39] Adedeji TA Adedeji NO Jeje OA Ajeigbe AK Smith OS Owojuyigbe TO . Serum reference intervals of micronutrients, vitamins, and interleukins among healthy adults in South-Western Nigeria. Pract Lab Med. (2024) 39:e00363. doi: 10.1016/j.plabm.2024.e00363. PMID: 38715661 PMC11075053

