# Supplementary material for: Reference intervals for plasma IFN-α, TNF-α, IL-12p70, and IFN-γ by flow cytometry in healthy adults from eastern China: a single-center study
Source: Front Immunol. 2026 May 14;17:1806685. doi: 10.3389/fimmu.2026.1806685 (PMC13215845; doi:10.3389/fimmu.2026.1806685)
Supplement: Supplementary file 1 [file Table1.doc]

**Supplemental Table 1** Performance Characteristics and Verification Results of Cytokine Assays

| Performance Parameters | Manufacturer’s specifications | Laboratory Verification Results | Conclusion  (Pass or fail) |
| --- | --- | --- | --- |
| LoB (pg/mL) | 0.20 pg/mL | 0.20 pg/mL | Pass |
| LoD (pg/mL) | 0.50 pg/mL | 0.50 pg/mL | Pass |
| LLoQ (pg/mL) | 1.00 pg/mL | 1.00 pg/mL | Pass* |
| Intra-assay precision | 8.00% | IFN-α：5.07% (Level 1) and 4.34% (Level 2)  TNF-α：6.42% (Level 1) and 3.47% (Level 2)  IL-12p70：2.71% (Level 1) and 3.24% (Level 2)  IFN-γ：5.46% (Level 1) and 4.60% (Level 2) | Pass** |
| Inter-assay precision | 12.00% | IFN-α：9.98% (Level 1) and 7.29% (Level 2)  TNF-α：6.76% (Level 1) and 5.54% (Level 2)  IL-12p70：3.84% (Level 1) and 4.23% (Level 2)  IFN-γ：7.65% (Level 1) and 6.03% (Level 2) | Pass** |
| Trueness | 15% | IFN-α：4.15% (Level 1) and 3.54% (Level 2)  TNF-α：7.69% (Level 1) and 2.05% (Level 2)  IL-12p70：5.12% (Level 1) and 6.75% (Level 2)  IFN-γ：2.87% (Level 1) and 6.55% (Level 2) | Pass |
| Linearity | 1.00-10,000.00 pg/mL  (R^2^≥0.995) | IFN-α：1.05-6,884.29 pg/mL (R^2^=0.999)  TNF-α：1.02-7,865.03 pg/mL (R^2^=0.999)  IL-12p70：1.09-7,595.14 pg/mL (R^2^=0.999)  IFN-γ：1.11-6,835.14 pg/mL (R^2^=0.998) | Pass*** |
| Reportable range | 1.00-20,000.00 pg/mL | IFN-α：1.05-13,768.58 pg/mL  TNF-α：1.02-15,730.06 pg/mL  IL-12p70：1.09-15,190.28 pg/mL  IFN-γ：1.11-13,760.28 pg/mL | Pass**** |

IFN-α: interferon-α; TNF-α: tumor necrosis factor-α; IL-12p70: interleukin-12p70; IFN-γ: interferon-γ; LoB: limit of blank; LoD: limit of detection; LLOQ: lower limit of quantification.

*Indicates that when cytokine concentrations are below the LLOQ (≤1.00 pg/mL), most healthy individuals show levels at or below the assay’s precise quantification threshold. Clinically, results reported as <LLOQ (<1.00 pg/mL) are considered within the expected healthy range, whereas quantifiable concentrations (≥1.00 pg/mL) may suggest the need for further evaluation of potential immune activation. Of note, the clinical utility of cytokines primarily lies in elevated levels, while the LLOQ has limited diagnostic or therapeutic value.

**Indicates that the validation was performed at two concentration levels: Level 1 (low concentration) and Level 2 (high concentration).

***Refers to cytokine concentrations verified in our laboratory using fresh samples with abnormally high values. Because samples matching the manufacturer’s declared high-value concentration (10,000.00 pg/mL) were unavailable, the verified concentrations are presented as shown in the table.

****Denotes the verified upper limits meet clinical application requirements. When concentrations exceed these limits, results are reported as > upper limit of detection (IFN-α > 13,768.58 pg/mL; TNF-α > 15,730.06 pg/mL; IL-12p70 > 15,190.28 pg/mL; IFN-γ > 13,760.28 pg/mL).

**Supplemental Table 2.** Conventional characteristics of reference individuals

| Indicators | All  (n=728) | Male  (n=364) | Female  (n=364) | Reference interval |
| --- | --- | --- | --- | --- |
| Age (years) | 48 (33, 60) | 48 (32, 58) | 47 (35, 62) | - |
| Smoking status*  (yes/no) | 265/472 | 157/207 | 108/256 | - |
| Alcohol consumption*  (yes/no) | 206/522 | 124/240 | 82/282 | - |
| BMI (kg/m^2^) | 23.2±2.3 | 23.5±2.0 | 22.8±2.6 | 18.5-27.9 |
| WBC (×10^9^/L) | 5.6±1.4 | 5.6±1.6 | 6.2±1.3 | 3.5-9.5 |
| CRP (mg/L) | 2.36 (1.06, 4.49) | 2.99 (1.52, 4.85) | 2.29 (1.23, 4.23) | 0.00-10.00 |
| ALT (U/L) | 23.9±7.7 | 25.3±7.1 | 22.4±8.0 | 9.0-50.0 (male)  7.0-40.0 (female) |
| AST (U/L) | 23.8±6.8 | 26.7±6.6 | 20.9±5.7 | 15.0-40.0 (male)  13.0-35.0 (female) |
| Urea (mmol/L) | 5.6±1.4 | 5.4±1.1 | 5.9±1.6 | 3.1-8.0 (male, aged 20-59 years)  3.6-9.5 (male, aged 60-79 years)  2.6-7.5 (female, aged 20-59 years)  3.1-8.8 (female, aged 60-79 years) |
| Crea (µmol/L) | 67±14 | 77±11 | 58±10 | 57-97 (male, aged 20-59 years)  57-111 (male, aged 60-79 years)  41-73 (female, aged 20-59 years)  41-81 (female, aged 60-79 years) |
| GLU (mmol/L) | 4.80±0.52 | 4.81±0.52 | 4.80±0.52 | 3.89-6.11 |
| TC (mmol/L) | 4.34±0.47 | 4.54±0.37 | 4.15±0.48 | <5.17 |
| TG (mmol/L) | 0.93±0.25 | 0.96±0.24 | 0.90±0.25 | <1.70 |

BMI: body mass index; WBC: white blood cell count; CRP: C-reactive protein; ALT: alanine aminotransferase; AST: aspartate aminotransferase; Urea: urea; Crea: creatinine; GLU: glucose;TC: total cholesterol; TG: triglyceride. The indicators of age and CRP were represented as the median (M) and interquartile range (IQR); the indicators of BMI, WBC, ALT, AST, Urea, Crea, GLU, TC and TG were represented by the mean ± standard deviation.

*Indicates that participants not meeting the inclusion criteria have been excluded.
